# Regulation of Matrix Metalloproteinase-2 Activity by COX-2-PGE2-pAKT Axis Promotes Angiogenesis in Endometriosis

**DOI:** 10.1371/journal.pone.0163540

**Published:** 2016-10-03

**Authors:** Sayantan Jana, Kasturi Chatterjee, Amlan K. Ray, Pramathes DasMahapatra, Snehasikta Swarnakar

**Affiliations:** 1 Cancer Biology & Inflammatory Disorder Division, CSIR-Indian Institute of Chemical Biology, Kolkata, West Bengal, India; 2 Spectrum Clinic & Endoscopy Research Institute, Kolkata, West Bengal, India; Medical College of Wisconsin, UNITED STATES

## Abstract

Endometriosis is characterized by the ectopic development of the endometrium which relies on angiogenesis. Although studies have identified the involvement of different matrix metalloproteinases (MMPs) in endometriosis, no study has yet investigated the role of MMP-2 in endometriosis-associated angiogenesis. The present study aims to understand the regulation of MMP-2 activity in endothelial cells and on angiogenesis during progression of ovarian endometriosis. Histological and biochemical data showed increased expressions of vascular endothelial growth factor (VEGF), VEGF receptor-2, cycloxygenase (COX)-2, von Willebrand factor along with angiogenesis during endometriosis progression. Women with endometriosis showed decreased MMP-2 activity in eutopic endometrium as compared to women without endometriosis. However, ectopic ovarian endometrioma showed significantly elevated MMP-2 activity with disease severity. In addition, increased MT1MMP and decreased tissue inhibitors of metalloproteinases (TIMP)-2 expressions were found in the late stages of endometriosis indicating more MMP-2 activation with disease progression. *In vitro* study using human endothelial cells showed that prostaglandin E2 (PGE2) significantly increased MMP-2 activity as well as tube formation. Inhibition of COX-2 and/or phosphorylated AKT suppressed MMP-2 activity and endothelial tube formation suggesting involvement of PGE2 in regulation of MMP-2 activity during angiogenesis. Moreover, specific inhibition of MMP-2 by chemical inhibitor significantly reduced cellular migration, invasion and tube formation. *In ovo* assay showed decreased angiogenic branching upon MMP-2 inhibition. Furthermore, a significant reduction of lesion numbers was observed upon inhibition of MMP-2 and COX-2 in mouse model of endometriosis. In conclusion, our study establishes the involvement of MMP-2 activity via COX-2-PGE2-pAKT axis in promoting angiogenesis during endometriosis progression.

## Introduction

Endometriosis is an invasive gynecological disorder of reproductive women characterized by the growth of endometrial glands and stroma outside the uterus. The disease is associated with chronic pelvic pain, severe dysmenorrhea, dyspareunia and infertility. Endometriosis affects almost 10–15% of women in reproductive age and 50% of women with infertility [1]. Although, it is believed to be an estrogen-dependent disease [2], the etiology and pathogenesis of endometriosis remains uncertain. According to the widely accepted ‘Sampson’s theory of retrograde menstruation’, endometriosis originates from the debris of endometrial glands. Detached endometrial tissues of menstruation which include endometrial cells, glands, debris etc, reach the peritoneum by retrograde movement to get implanted, followed by acquisition of new blood supply through angiogenesis [3]. Endometriotic growths are supported by the local hormonal and inflammatory microenvironment and further spread over multiple locations within the peritoneum.

Pathological angiogenesis is the hallmark of many diseases including endometriosis [4]. Angiogenesis occurs in a complex dynamic mechanism which starts with destabilization of mature blood vessel through detachment of mural cells and degradation of extracellular matrix (ECM). The exposed specialized endothelial ‘tip cells’ starts budding and spouting upon pro-angiogenic stimulus from local environment [5]. The endothelial cells located behind the migrating endothelium of the sprouts reproduce and constitute a structured tunnel of endothelial cells for the developing blood vessels. Newly formed blood vessels then stabilize upon recruitment of pericytes and smooth muscle cells followed by production of ECM components over the vessels [5]. The pro-angiogenic milieu in the peritoneum of endometriosis patients supports the growth of the ectopic implants. Endometriosis patients show increased levels of vascular endothelial growth factor (VEGF) in the ectopic tissues and peritoneal fluids [6, 7]. VEGF is produced by many cells including stromal, endothelial, neutrophil etc. However, steroid-mediated regulation of macrophages is important for increased VEGF levels and is attributed to the VEGF-dependent elevated endothelial cell proliferation in women with endometriosis [8]. Endometriosis patients also show increased levels of other angiogenic factors including IL-8, hepatocyte growth factor (HGF), erythropoietin, angiogenin, macrophage migration inhibitory factor, neutrophil-activating factor and tumor nacrosis factor (TNF)-α which promote angiogenesis during endometriosis progression [9].

Because matrix metalloproteinases (MMPs) are essential in orchestrating proper physiological functioning of the endometrium; hence, alteration of MMP activities is considered as a critical factor for the development of endometriosis. MMPs are a group of zinc-dependent proteolytic enzymes that are mainly involved in ECM degradation to promote cellular invasion, migration and events like angiogenesis [10, 11]. MMPs are also involved in the cellular event of epithelial-mesenchymal transition [12]. Majority of the MMPs are secreted as latent pro-enzyme form and activated through the proteolytic cleavage of the pro-domain. MMP activities are regulated by their endogenous inhibitors, tissue inhibitors of metalloproteinases(TIMPs) [13]. The roles of MMPs in endometriosis are intriguing; there are reports for the presence or elevated expressions of MMP-9, -2, -3 and -7 in human endometriosis [14–16]. Studies from our laboratory as well as others have shown that eutopic endometrium of women with endometriosis exhibits higher MMP-9 activities than unaffected women [15]. Studies also demonstrated the increased MMP-3 expressions in the ectopic endometrial tissues of induced endometriosis [16, 17]. Suppression of MMPs was reported to inhibit the establishment of ectopic lesions from human endometrium in nude mice [18].

MMP-2, also known as gelatinase A, facilitates cancer cell invasion and metastasis by degrading collagen IV, V and X which are present in the ECM and basement membrane [19]. MMP-2 is also involved in integrin αVβ3-mediated signaling in cancer cells [20]. In addition, MMP-2 expression was elevated during the proliferative phase of the menstrual cycle. Peritoneal fluids and serum of endometriosis patients contained higher levels of MMP-2 than those of unaffected patients [21]. Moreover, MMP-2 expression in the peritoneal fluid of endometriosis patients was positively correlated with 17β-estradiol level, and negatively correlated with progesterone levels [22]. MMP-2 is secreted as proMMP-2 and is later activated through TIMP-2 and MT1MMP-dependent mechanisms [23]. The MT1MMP null mouse has impaired endochondral ossification and angiogenic defects due to attenuated MMP-2 activity during development [24]. The present study has evaluated the angiogenic role of MMP-2 activity in ovarian endometriosis patients and looked into the signaling responses to PGE2, using *in vivo* and *in vitro* studies.

## Material and Methods

### Chemicals

Gelatin, Triton X-100, chemical inhibitors (GM6001, ARP101, NS398, AKT kinase inhibitor), prostaglandin E2, celecoxib, protease inhibitors mixture, 5-bromo-4-chloro-3-indolyl phosphate/nitro blue tetrazolium and chemical inhibitors were obtained from Sigma Aldrich Inc, St. Louis, MO, USA. Pre-stained protein molecular weight markers were purchased from Fermentas Inc, Washington, DC, USA. Antibodies were obtained from Santa Cruz Biotechnology Inc, California, USA (S1 Table). All other chemicals were purchased from Sisco Research Laboratories, Mumbai, India.

### Human Study

Serum and ectopic samples were collected from 88 women with endometriosis-associated complications, attending the gynecology unit of Spectrum Clinic & Endoscopy Research Institute, Kolkata, India. The study protocol was approved by the Human Ethics Committee of Indian Institute of Chemical Biology and Human Ethics Committee of Spectrum Clinic & Endoscopy Research Institute, Kolkata, India. All participants gave written informed consent for participation. The clinical diagnosis of ovarian endometriosis was confirmed by laparoscopy and all serum and biopsy samples were collected during proliferative phase of the menstrual cycle. Control samples (n = 15) were collected from women who were undergoing laproscopic surgery, exhibited no visible evidence of endometriosis upon laparoscopy. A detailed demographic profile of the study population is given in Table 1. Briefly, we collected four categories of ovarian endometriosis samples from stage I-IV (stage-I, 19; stage-II, 14; Stage-III, 24; and stage-IV, 31 samples). The stages of endometriosis were indexed according to the score of revised American Society for Reproductive Medicine (rASRM). After collection, all serum and biopsies were stored at -80°C for future experiments.

**Table 1:** Demographic profile of the study population.

	Control	Endometriosis Stage I	Endometriosis Stage II	Endometriosis Stage III	Endometriosis Stage IV
Population (N)	15	19	14	24	31
Age ± SEM	32±6.7	34.89±7.63	31.5±6.04	32.58±6.40	32.54±5.4
Infertility (%)	2/15(13.33)	11/19(57.89)	12/14(85.71)	18/24(75)	21/31(67.74)
Primary Infertility (%)	1/15(6.66)	7/19(36.84)	8/14(57.14)	16/24(66.66)	16/31(51.61)
Secondary Infertility (%)	1/15(6.66)	4/19(21.05)	6/14(42.85)	2/24(8.33)	5/31(16.12)
Dysmenorrhea (%)	3/15(20)	8/19(42.10)	10/14(71.42)	17/24(70.83)	21/31(67.74)

Control (n = 15) and endometriosis samples (n = 88) on the basis of severity (as standardized by rASRM) were collected and grouped as stage I, II, III and IV. All individuals of the study population were in the proliferative phase of the menstrual cycle during the study.

### Animal experiment

Female adult BALB/c mice of 6–8 weeks old, bred in house with free access to food and water were used in all experiments. Animal experiments were approved and carried out following the guidelines of the Animal Ethics Committee of CSIR-Indian Institute of Chemical Biology, Kolkata, India. Induction of peritoneal endometriosis was performed as reported previously [15]. Briefly, on day 0 the donor mice were anesthetized (ketamine 12 mg/kg body weight) and sacrificed to obtain uterine horns under sterile conditions. The endometrium was carefully teased out and suspended in 0.6 ml of sterile phosphate buffer saline (PBS) and inoculated into the peritoneal cavity of recipient mice containing subcutaneous implants of estradiol-17β (25 μg/ml) pellet with a ratio of one donor to two recipients. Among three endometriosis groups (n = 4 for each group), two groups were administered separately with MMP-2i (ARP101) and COX-2i (celecoxib) intraperitoneally (i.p.) 20mg/kg and 40mg/kg b.w. respectively once every alternate day for the period of 10 days. Only endometriosis (vehicle) group was treated with DMSO. Animals were anesthetized by ketamine (12 mg/kg b.w.) and sacrificed by cervical dislocation on day 10 post-induction of endometriosis and visible endometriotic lesions (more than 2 mm in diameter) were counted and preserved for further experiments.

### In vitro study

Human umbilical vein endothelial cell (HUVEC, catalogue C0035C) was purchased from Invitrogen (Thermo Fisher Scientific Corporation, Massachusetts, USA). MDAMB-231 breast cancer cells were procured from NCCS, Pune, India. MDAMB-231 cells were grown in plastic cell culture dishes in 95% air/5% CO_2_ in DMEM supplied with 20 mM HEPES, 10% heat-inactivated FBS, 2 mM-glutamine, 100 U/mL penicillin, and 100 μg/mL streptomycin. Endothelial cells cultured in LSGS-supplemented 200PRF Medium (Invitrogen). Inhibition of MMP-2 (ARP101) or broad spectrum MMPs (GM6001) was performed in MDAMB-231(12.5μM) for 24 h for migration and invasion experiments. HUVEC cells were treated with prostaglandin E2 (0.1–1μM/L) for 6 h in non-supplemented 200PRF medium. Cell supernatant was subjected to gelatin zymography and total cell extract was subjected to immunoblotting.

### Cell and tissue extraction

Tissues were suspended in PBS containing protease inhibitors, minced at 4°C. The suspension was centrifuged at 12,000 g for 15 min, and supernatant was collected as PBS extracts. The pellet was further, extracted in lysis buffer (10 mM Tris-HCl, pH 8.0, 150 mM NaCl, 1% Triton X-100, and protease inhibitors) and centrifuged at 12,000 g for 15 min to obtain Triton X-100 (Tx) extracts. *In vitro* cells were directly homogenized in lysis buffer containing protease inhibitor cocktail and centrifuged at 12,000 g for 15 min to obtain the whole cell extract. Proteins were estimated either by Lowry method or Bradford assay.

### Migration assay

For migration assay, MDAMB-231 cells were cultured to confluence and scratched with a pipette tip to a constant diameter (marked in dashed line). Cells were treated with 12.5μM of broad spectrum MMP inhibitor (GM6001), MMP-2 inhibitor (ARP101) and after 24 h images were captured in Olympus microscope using Camedia software (Chicago, MI, USA) (E-20P 5.0 megapixel) and processed using Adobe Photoshop version 7.0(Adobe Systems Incorporated, San Jose, CA, USA). Cells were counted from four random sites for each group and experiment was repeated at least three times independently. Data represented as the average of counts ± SE.

### Invasion assay

The invasion assays were performed with transwell Boyden chamber assay kit (BD Biosciences, MA, USA), using MDAMB231 cells. 25,000 cells were added to upper chamber of the well with or without MMP/MMP-2 inhibitors (12.5μM) in a serum free condition. The lower chamber contained complete media (with 10% FBS). After 24 h, cells from the upper chamber were removed and lower chamber cells were fixed and stained with 1% crystal violate. Cells were counted from 4 random sites for each well and experiment was repeated at least three times independently. Images were captured in Olympus microscope using Camedia software (Chicago, MI, USA) (E-20P 5.0 megapixel) and processed using Adobe Photoshop version 7.0. Data are represented as the average of counts ± SE.

### Chorioallantoic membrane assay

Day 0 fertilized eggs of white leghorn chickens were kept at 37°C under sterile conditions. After 7 days, human endometriosis extract (stage IV, 500μg) with or without MMP/MMP-2 inhibitor (25μM) impregnated in gelatin discs were implanted in the CAM models through a 1 cm^2^ window made on the shells of the eggs (n = 4). Control eggs were implanted with vehicle-impregnated discs in an identical manner. After resealing, eggs were incubated at 37°C in a humidified chamber for 48 h, and then opened and observed macroscopically. Quantitative measurements of the branching points were performed after capturing photographs and were computed using Adobe Photoshop 7. The dashed circle within CAM indicates the zone of quantification and asterix indicates the site of disc implantation.

### Matrigel assay

Matrigel assay was performed using HUVEC cells. Endothelial cells cultured in LSGS-supplemented 200PRF Medium (Invitrogen, Thermo Fisher Scientific Corporation, Massachusetts, USA). Prior to cell inoculation, 100 μl matrigel (geltrex matrix, Invitrogen) was coated on 96 well plate to solidify and 3x10^4^ cells/well were seeded in non-supplemented 200PRF medium with or without inhibitors (5μM for Akt1/2 kinase inhibitor, COX-2 inhibitor-NS398, MMP-2 inhibitor-ARP101, broad spectrum MMP inhibitor-GM6001)/stimulators (prostaglandinE2-1μM). After 4 h of cell seeding, tube formation was quantified using Olympus microscope, Camedia software and processed using Adobe Photoshop version 7.0 (Adobe Systems Incorporated, San Jose, CA, USA). Tube formations were counted from 4 random sites for each well and the experiment was repeated at least three times independently. Data represented as the average tube formation of field counts ± SE.

### Gelatin Zymography

For assay of MMP-2,-9 activity, extracts (30 μg protein/lane) or serum (2μl/lane) or cell supernatant (10μl/lane) were electrophoresed in 8% SDS-polyacrylamide gel containing 1 mg/ml gelatin under non-reducing conditions. The gels were washed twice in 2.5% Triton X-100 and then incubated in calcium assay buffer (40 mM Tris-HCl, pH 7.4, 0.2 M NaCl, 10 mM CaCl_2_) for 18 h at 37°C. Gels were stained with 0.1% Coomassie blue followed by destaining. The zones of gelatinolytic activities appeared as negative staining. Quantification of zymographic bands was done using densitometry linked to proper software (Lab Image, Kapelan Gmbh, Leipzig, Germany).

### Hematoxylin & Eosin staining

Tissues were sectioned into 2–3 mm^2^ pieces. The tissue samples were fixed in 4% paraformaldehyde, dehydrated and embedded in paraffin wax. Approximately, 5μm thick serial sections were rehydrated in descending alcohol series and stained with hematoxylin and eosin. Fixation, permeabilization, and staining runs were carried out in exact parallel to ensure comparative significance among groups. Images were captured in Olympus microscope using Camedia software (Chicago, MI, USA) and processed using Adobe Photoshop version 7.0.

### Immunostaining

Deparaffinised and rehydrated sections were subjected to antigen retrieval by trypsin (0.05% trypsin, 0.1% CaCl_2_) and blocking was performed using 5% BSA in TBS (20 mM TrisHCl, pH 7.4 containing 150 mM NaCl) for 2 h at room temperature followed by the incubation over night at 4°C in primary antibody solutions (1:100 dilutions in TBS with 1% BSA) in a humid chamber. The tissue sections were washed four times with TBST (20 mM TrisHCl, pH 7.4 containing 150 mM NaCl and 0.025% Triton X-100) followed by incubation with texus red secondary antibody (Santa Cruz Biotechnology, USA) solution (1:200 dilutions in TBS containing 1% BSA) for 2 h at room temperature. Tissue sections were washed four times with TBST followed by counter staining with DAPI. For IHC, horse reddish peroxidase-conjugated secondary antibodies (1:400 dilutions) were used. Haemotoxylin was used as counter staining and DAB was used as substrate to develop colour. The images were observed in fluorescence Olympus microscope and images were captured using Camedia software (E- 20P 5.0 Megapixel) and processed under Adobe Photoshop version 7.0.

### Western blotting

Tissue (100 μg) or cell (40μg) extracts were resolved by 10% reducing SDS-polyacrylamide gel electrophoresis and transferred to nitrocellulose membranes. The membranes were blocked for 2 h at room temperature in 3% BSA solution in 20 mM Tris-HCl, pH 7.4 containing 150 mM NaCl and 0.02% Tween 20 (TBST) followed by overnight incubation at 4°C in 1:500 dilution of the respective primary antibodies in TBST containing 0.2% BSA. The membranes were washed five times with TBST and then incubated with alkaline phosphatase-conjugated secondary antibody (1:10,000 dilution). The bands were visualized using 5-bromo-4-chloro-3-indolyl phosphate/nitro blue tetrazolium substrate solution.

### Statistical analysis

Experiments were repeated for at least three times independently. Protein band intensities were quantified by densitometric analysis using Lab image (version 2.7.1, Kapelan GmbH, Germany) software. The statistical analysis of the data was done using GraphPad Instat-3 (version 3.06, San Diego, California, USA) software. Comparison between groups was performed using one-way analysis of variance (ANOVA) followed by Student–Newman–Keuls T-test. Data were fitted using Sigma plot (version11.0, GmbH, Germany) represented as means ± SEM. p < 0.05 was accepted as level of significance; *** very highly significant p < 0.001; ** highly significant p < 0.01; * significant p < 0.05; NS not significant for p > 0.05.

## Results

A total of 88 ovarian endometriosis and 15 control samples were analyzed during the study. The ovarian endometriomas were grouped in four categories based on the severity of the disease e.g. stage I-IV (stage-I, 19; stage-II, 14; Stage-III, 24; and stage-IV, 31 samples). Infertility and dysmenorrheal status of the population was illustrated in Table 1.

### Ovarian endometriosis is associated with angiogenesis

**Fig 1:**
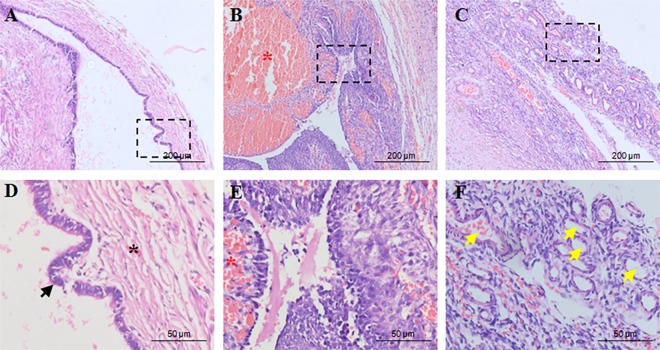
Characteristics of ectopic ovarian endometriosis. Hematoxylin and Eosin staining of ectopic endometriosis show presence of (A,D) endometrial glands (black arrow) and stroma(black asterisk), (B,E) hemorrhage and iron deposition (red asterisk) and (C,F) aberrant blood vessel formation(yellow arrow). The dashed box indicates the magnified area in the lower panel.

**Fig 2:**
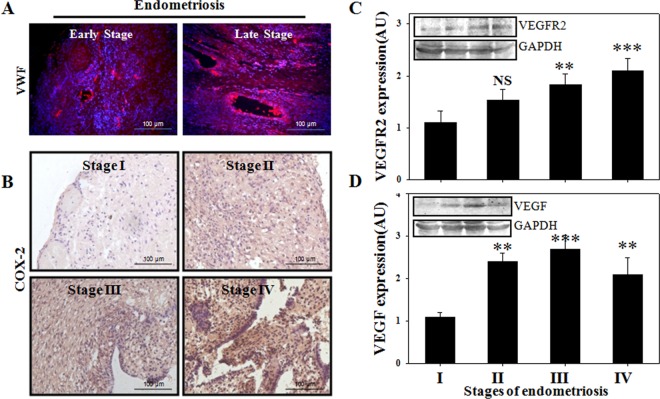
Involvement of angiogenesis in ovarian endometriosis. (A) Immunofluorescence for Von Willebrand factor (red flourescence) in early and late stages (n = 3 samples/stage) of ovarian endometriosis. Nucleus was stained with DAPI (blue flourescence). (B) Immunohistochemistry for cycloxygenase-2 in different stages (n = 3 samples/stage) of endometriosis. (C-D) Quantification of VEGF receptor-2(n = 14 samples/stage) and VEGF expressions (n = 12 samples/stage) in ectopic samples of endometriosis. Means ± SEM. p < 0.05 was accepted as level of significance; *** p < 0.001; ** p < 0.01; * p < 0.05; NS p > 0.05.

Since the implantation of endometrial fragments at foreign sites requires neo-vascularization, it seems plausible that angiogenesis plays a pivotal role for the growth of these ectopic implants. To explore whether ovarian endometrioma undergoes angiogenesis, we performed histological studies on the ectopic tissues. Hematoxylin & Eosin staining of human ovarian endometriosis showed development of endometriotic glands and stroma (Fig 1A and 1D), hemorrhage and iron deposition (Fig 1B and 1E). Furthermore, the sections of ovarian endometriomas exhibited aberrant blood vessel formation indicating angiogenesis at these ectopic tissues (Fig 1C and 1F). To confirm neo-vascularisation, we performed immunofluorescence study with anti-von Willebrand factor (VWF) antibody in the early and late stages of ovarian endometriosis. VWF was found to be specific to blood vessels and showed increased expression at the late stages of the disease (Fig 2A). Since COX-2 is an important regulator of angiogenic milieu, we also checked its expressions by immunohistochemistry in different stages of the disease. COX-2 expression was significantly elevated and was found to be localized both in the glandular epithelium and stromal cells (Fig 2B and S1 Fig). VEGF is an indispensible factor for angiogenesis, as it regulates endothelial cell proliferation, sprouting, assembly, lumen development, permeability and distribution of the vessels. To assess the expressions for VEGF and VEGFR2 immunoblottings were performed with ovarian endometriomas in a severity dependent manner (Fig 2C and 2D and S1 Fig). The expressions for both VEGF and VEGFR2 were significantly increased with the advancement of the disease, confirming the involvement of angiogenesis during ovarian endometriosis progression.

### Activity of MMP-2 in eutopic endometrium of women with and without endometriosis

**Fig 3:**
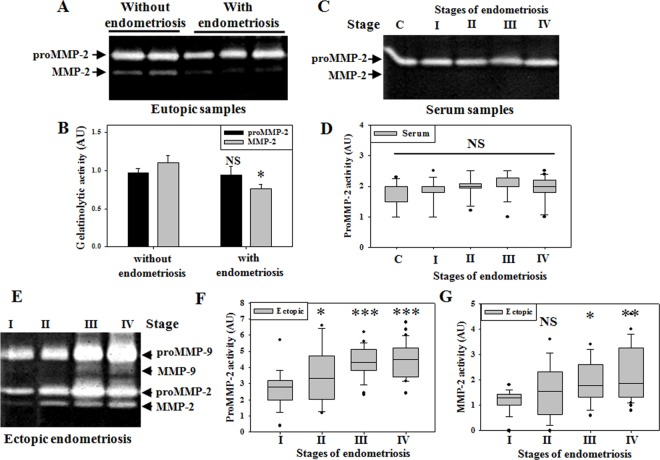
Involvement of MMP-2 activity in ovarian endometriosis. (A,B) Status of MMP-2 activity in eutopic endometrium (n = 15 samples/group) between women with and without endometriosis. (C,D) Serum MMP-2 status in different stages of ovarian endometriosis patients. (E-G) Status of MMP-2 activity in ectopic ovarian endometriotic tissues. (F,G) Representative zymography profile for pro and active MMP-2 in ectopic ovarian endometriosis. Number of patients for stage I (n = 19), stage II (n = 14), stage III (n = 24), stage IV (n = 31). Means ± SEM. p<0.05 was accepted as level of significance; *** p < 0.001; ** p < 0.01; * p < 0.05; NS p > 0.05.

Since endometriosis influences endometrium receptivity [25] and MMP-9 responses [15], we explored whether women with endometriosis are predisposed to change in the activity of MMP-2 in eutopic endometrium. To assess the activity of MMP-2, gelatin zymography was performed (Fig 3A and S2 Fig). We found that the eutopic endometrium contained both pro and active forms of MMP-2. Women with endometriosis showed significantly reduced level of active MMP-2 in eutopic endometrium as compared to women without endometriosis. No significant changes were detected for proMMP-2 activities in eutopic samples between women with and without endometriosis (Fig 3B).

### Severity dependent MMP-2 activity in ovarian endometriomas

Although several studies have reported presence of MMP-2 in endometriosis [21, 22]; no study has looked into MMP-2 activity during pathogenesis of endometriosis. To evaluate the activity of MMP-2, gelatin zymography was performed with serum samples obtained from women without endometriosis (control) and patients with varying stages of ovarian endometriosis (Fig 3C and S2 Fig). We found that serum contains only pro-form of MMP-2 and no significant difference of proMMP-2 activity was observed for different stages of endometriosis in comparison to control samples (Fig 3D). However, unlike serum, both pro and active forms of MMP-2 were present in ectopic samples of ovarian endometriosis. Both proMMP-2 and active MMP-2 activities were significantly elevated with the progression of the disease, which also suggested increased pro-to-active form transition of MMP-2(Fig 3E–3G). We also found elevated MMP-9 activities in the ectopic endometriosis in a stage dependent manner, which corroborated our previous report [15].

### Involvement of MT1MMP and TIMP-2 in MMP-2 activation during progression of endometriosis

**Fig 4:**
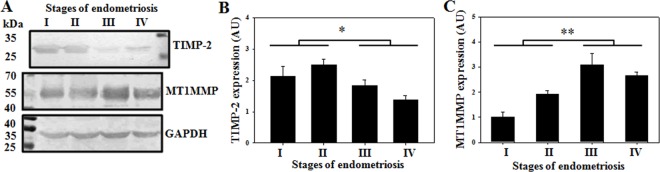
Status of TIMP-2 and MT1MMP expressions in ovarian endometriosis ectopic samples. Representative western blots (A) and profiles for expressions of TIMP-2 (n = 14 samples/stage) and MT1MMP (n = 12 samples/stage) in ectopic ovarian endometriosis(B,C). Means ± SEM. p<0.05 was accepted as level of significance; *** p < 0.001; ** p < 0.01; * p < 0.05; NS p > 0.05.

Several proteolytic enzymes are involved during pro-to-active transition of MMPs [26]. However, for MMP-2 activation, TIMP-2 and MT1MMP (membrane type MMP-1/MMP-14) play important roles. Usually, TIMP-2 acts as an endogenous inhibitor for MMP-2 by interacting with its N-terminal domain. However, if the N-terminal domain of TIMP-2 is preoccupied with the insoluble MT1MMP, the C-terminal domain interacts with the proMMP-2. This event opens up an opportunity for the nearby free MT1MMP to cleave the pro-domain of MMP-2, releasing the active protein [23]. To understand whether the same mechanism is also involved with endometriosis, TIMP-2 and MT1MMP expressions in ectopic samples were quantified by immunoblotting (Fig 4 and S1 Fig). Our results showed that while TIMP-2 expression decreased with disease severity (Fig 4A and 4B), MT1MMP expression elevated with the progression of the disease (Fig 4A and 4C). The reduced expression of TIMP-2 indicated attenuated inhibition on MMP-2 activity, however it also suggested that limited TIMP-2 and increased MT1MMP expressions are associated with increased MMP-2 activation during the progression of endometriosis.

### ProstaglandinE2 promotes MMP-2 in human endothelial cells

**Fig 5:**
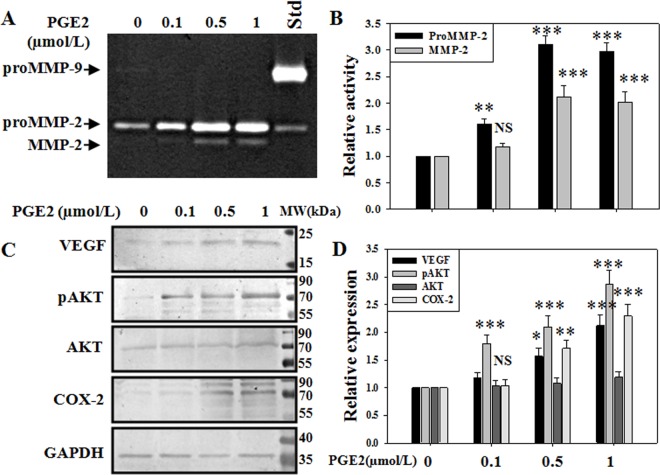
Effects of prostaglandin E2 on MMP-2 activity in endothelial cells. (A,B) HUVEC cells were treated with PGE2 (0.1–1μM) for 6hr and gelatin zymography was performed with cell supernatant. (C,D) Whole cell extract (40μg/lane) were immunoblotted for VEGF, pAKT, AKT, COX-2. Experiment was repeated at least three times independently. *** p < 0.001; ** p < 0.01; * p < 0.05; NS p > 0.05.

Although patients with endometriosis were reported to have elevated PGE2 levels [27, 28], the effect of PGE2 on MMP-2 is still not well investigated. To understand that, human umbilical vein endothelial cells (HUVEC) were treated with increasing doses of PGE2 (0.1–1μM) and the MMP-2 activity were assessed through gelatin zymography (Fig 5 and S3 Fig). Our results revealed that PGE2 increased both the pro and active MMP-2 activities in a dose dependent manner. Moreover, when immunoblotting was performed, we found that PGE2 treatment elevated the expressions of VEGF, phosphorylated AKT (pAKT), and COX-2 in endothelial cells (Fig 5C and 5D). The current data suggested that the PGE2-mediated elevation of MMP-2 activity is associated with the pro-angiogenic responses.

#### Inhibition of COX-2-PGE2-pAKT axis attenuates MMP-2 activity and tube formation in endothelial cells

**Fig 6:**
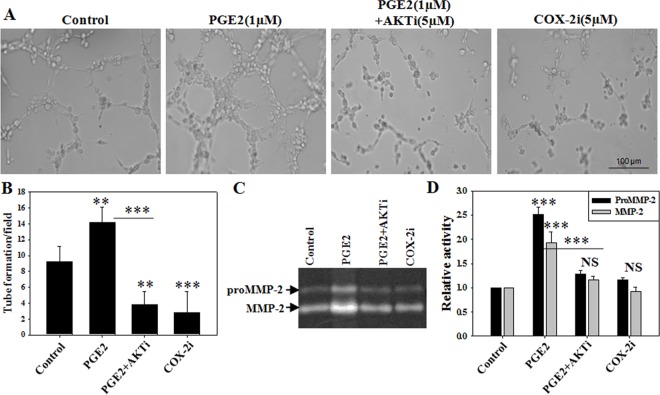
Inhibition of prostaglandinE2 attenuates tube formation along with MMP-2 activity in endothelial cells. (A,B) HUVEC cells were inoculated on matrigel in the presence of PGE2 (1μM) with or without AKT1/2 kinase inhibitor (5μM), or only COX-2 inhibitor (5μM) and tube formation was monitored. (C,D) Gelatin zymography was performed to assess MMP-2 activity from cell supernatant. Experiments were repeated for at least three times independently. *** p < 0.001; ** p < 0.01; * p < 0.05; NS p > 0.05.

To explore whether PGE2 mediated MMP-2 activity was also involved in endothelial tube formation, an important event for angiogenesis, we performed matrigel assay using endothelial cells with or without PGE2 treatment. We found that PGE2 treatment significantly increased tube formation of endothelial cells (Fig 6A and S3 Fig) than vehicle treatment. To further explore the related pathway, we performed tube formation assay in presence of AKT kinase inhibitor with PGE2 and COX-2 inhibitor without PGE2. Interestingly, even in presence of PGE2, inhibition of pAKT significantly reduced tube formation in comparison to only PGE2 treatment. Gelatin zymography with cell supernatant showed that PGE2-mediated elevated MMP-2 activity was involved with increased endothelial tube formation. Moreover, inhibition of AKT kinase significantly attenuated MMP-2 activity, indicating pAKT as downstream regulator of PGE2 signaling pathways. PGE2 mediated MMP-2 activity was revalidated with inhibition of upstream COX-2, which significantly reduced MMP-2 activity as well as tube formation of the endothelial cells (Fig 6A–6D).

### Role of MMP-2 in cellular invasion, tube formation and angiogenesis

**Fig 7:**
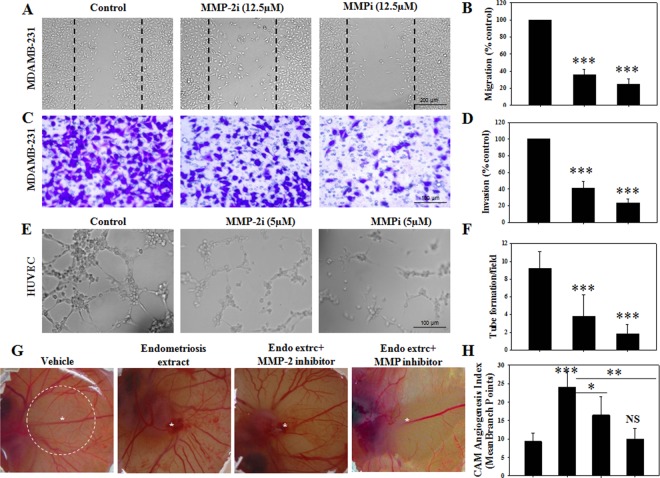
Role of MMP-2 on cellular invasion and angiogenesis. (A,B) Migration and (C,D) invasion assay was performed on MDAMB-231 cells with or without inhibitors (MMP-2i, 12.5μM or MMPi, 12.5μM). (E,F) Tube formation using HUVEC cells was performed on matrigel in the presence or absence of inhibitors (MMP-2i, 5μM or MMPi, 5μM). (G,H) Angiogenesis assay (chick chorioallantoic membrane assay) was performed using human endometriosis extract (500μg/implant) with or without MMP inhibitors (MMP-2i, 25μM or MMPi, 25μM). Dashed area signifies zone of quantification. Experiments were repeated for at least three times independently. *** p < 0.001; ** p < 0.01; * p < 0.05; NS p > 0.05.

To explore the role of MMP-2 activity on cellular behavior, MDAMB-231 cells were treated with either specific MMP-2 inhibitor or broad spectrum MMP inhibitor and cellular migration and invasion assays were performed (Fig 7A–7D). We found that inhibition of MMP-2 activity significantly attenuated cellular migration (Fig 7A and 7B). Moreover, transwell migration/invasion assay, which relies on secreted MMPs to cleave the basement ECM for cellular invasion towards the basal surface, showed significant attenuated cellular invasion upon MMP-2 inhibition (Fig 7C and 7D). Treatment with broad spectrum MMP inhibitor also reduced cellular invasion and migration significantly in comparison to vehicle treatment. The current results indicate that MMP-2 alone has competent roles for regulating cellular migration and invasion.

To explore whether the absence of only MMP-2 activity can influence endothelial tube formation, matrigel assay was performed in presence of specific MMP-2 inhibitor or broad spectrum MMP inhibitor (Fig 7E and 7F). Inhibition of MMP-2 in matrigel assay significantly reduced tube formation as compared to vehicle treatment. Inhibition of broad spectrum MMPs, however, showed further inhibitory effects for endothelial tube formation (Fig 7E and 7F). Finally, to understand the angiogenic potential of MMP-2 in endometriosis, chick chorioallantoic membrane (CAM) assay was performed. Human endometriosis extract (500μg/implant) was implanted for each CAM model as source for pro-angiogenic factors. As expected, CAM impregnated with only human endometriosis extract exhibited significantly elevated angiogenic branching implying increased angiogenesis than vehicle treatment. However, when the same sample was inoculated with selective inhibitor for MMP-2, angiogenic branching was significantly reduced in comparison to only endometriosis extract treatment (Fig 7G and 7H). Moreover, broad spectrum MMP inhibitor in the same experiment showed more effective inhibitory actions on CAM angiogenesis. The current data elucidated the importance of MMP-2 activity on cellular invasion, migration, and subsequent roles for endothelial tube formation and angiogenesis in CAM model.

### Effect of inhibition of MMP-2 and COX-2 in mouse model of endometriosis

**Fig 8:**
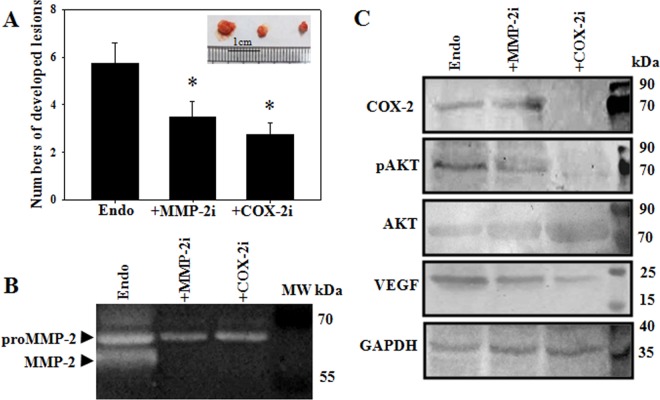
Effect for inhibition of MMP-2 and PGE2 in mouse model of endometriosis. Mouse model of endometriosis were developed by inoculation of endometrial cells into peritoneum of the recipient mice as described in the ‘material and methods’. MMP-2i (20mg/kg) and COX-2i (40mg/kg) were administrated every alternate day for 10days. Mice (n = 4 for each group) were sacrificed on day 10 from the day of endometriosis induction. (A) Histogram for numbers of developed lesions in mice peritoneum. Inset shows the developed lesions. (B) Gelatin zymography was performed for MMP-2 activities of endometriosis. (C) Western blot was performed with the whole tissue lysate for mouse model of endometriosis. Means ± SEM. p < 0.05 was accepted as level of significance; * p < 0.05.

To evaluate the potential of MMP-2 inhibition on endometriosis and angiogenesis, mouse model of endometriosis was performed and treated with MMP-2i (20mg/kg) and COX-2i (celecoxib 40mg/kg) during the development of endometriotic lesions. In comparison to the vehicle treated endometriosis lesions, MMP-2i treated mice developed significantly reduced numbers of endometriosis lesions (Fig 8A and S4 Fig). Endometriotic lesions showed elevated active MMP-2 activities and VEGF expressions, which were significantly decreased upon MMP-2i treatments (Fig 8B and 8C). Treatment with celecoxib, which is a selective inhibitor of COX-2 and thus also inhibit downstream PGE2, reduced the numbers of developed endometriotic lesions significantly (Fig 8A). Moreover, COX-2i significantly reduced MMP-2 activities, along with VEGF expressions inhibiting angiogenesis. Treatment with COX-2i also reduced pAKT expressions, while the total AKT level remained elevated (Fig 8C), suggesting that COX-2 inhibition attenuated phosphorylation of AKT.

## Discussion

Angiogenesis, defined as the formation of new blood vessels from preexisting blood vessel, is a physiological response but can also attribute to pathological conditions including cancer and endometriosis. Presence of increased vessel density is one of the typical characteristics of endometriosis and our histological findings support the fact. We report the presence of aberrant blood vessels in the ectopic endometriomas. Endometriosis patients show elevated levels of different pro-angiogenic factors including IL-8, IL-6, angiogenin, HGF and prostaglandin E2 [9]. The peritoneal fluid and serum of the endometriosis patients contained higher levels of VEGF in comparison to control women [7]. In accordance, our results show increased ectopic expressions for VEGF and VEGFR2 with advancement of the disease. The importance of VEGF in pathological angiogenesis is immense, as it governs almost every step of the process including endothelial cell proliferation, sprouting, assembly, lumen development, permeability and pattern distribution for vessels[29]. Moreover, VEGF is reported to act on the endothelial cells mainly through VEGFR2 mediated pathway to promote the early angiogenic responses [30]. Our results show increased VEGF and VEGFR2 in the ectopic tissues indicating neo-vascularization in endometriosis. We also found expressions of von Willebrand factor which is a marker for angiogenesis elevated with progression of endometriosis. Moreover, COX-2, a key enzyme for PGE2 synthesis from arachidonic acid, is increased with the disease severity. Our data show higher expressions of COX-2 in stromal and glandular cells of endometriosis which corroborates with other reports[31].

Herein, we address the importance of MMP-2 in relation to pathological angiogenesis during progression of endometriosis. MMP-2 is usually expressed in a wide range of cells and is believed to maintain basic functions for ECM remodeling. Thus, both reduced and elevated activities of MMP-2 may result in abnormal physiological functions. The present study documents reduced MMP-2 activities in uterine endometrium of the endometriosis patients than the women without endometriosis. The endometrium requires hormone-dependent periodic remodeling and angiogenic responses for its proper functioning; thus attenuated MMP-2 activity might indicate abnormal cellular and angiogenic responses in the uterus of endometriosis patients. However, in ectopic ovarian endometriosis, increased MMP-2 activities were observed with the disease progression, suggesting its involvement in the pathogenesis through aberrant cellular remodeling. Apparently, the increased MMP-2 activity was restricted to ectopic tissues only, influencing the local microenvironment and angiogenic milieu. We report increased MT1MMP and decreased TIMP-2 expressions with the disease severity, implying not only reduced inhibition of TIMP-2 over MMP-2 activity but also increased activation of MMP-2 during the late stages of the disease. In accordance, previous study from our laboratory has found reduced interaction between TIMP-2 and MMP-2 during MMP-2 activation in mouse model of early endometriosis [32]. TIMP-2 acts for both activation and inhibition of MMP-2 [32, 33]; however TIMP-2 also exhibits MMP-independent angiogenesis responses through inhibition of endothelial cell proliferation[34]. Activation of MMP-2 also relies on MT1MMP; MT1MMP null mice manifest impaired endochondral ossification and angiogenic defects due to attenuated MMP-2 activity during development and die within 3 weeks of age [24].

Literature revealed that the women with endometriosis exhibit higher levels of PGE2 in peritoneal fluids than the women without endometriosis [27, 28]. Furthermore, PGE2 level in peritoneal fluids of endometriosis patients significantly elevate at the late stages of endometriosis in comparison to early stages [21]. PGE2-mediated responses is also associated with estrogen levels in endometriosis [28]. The present study documents PGE2-dependent increase of MMP-2 activity in human endothelial cells. In accordance, positive correlation of MMP-2 with 17β-E2 levels in the peritoneal fluids of endometriosis patients has been documented [22]. Attenuation of MMP-2 activity and tube formation by COX-2i treatment further confirmed the regulation of MMP-2 activity via PGE2. Moreover, inhibition of downstream pAKT decreased MMP-2 activity and tube formation in HUVEC cells, indicating that the PGE2-mediated regulation of MMP-2 activity and endothelial tube formation occurs through pAKT pathway. These results are further supported by the fact of selective inhibition of MMP-2 during *in vitro* assays of cellular migration and invasion. Furthermore, reduced tube formation of endothelial cells by inhibiting MMP-2 activity directly proves its relevance on angiogenesis.

**Fig 9:**
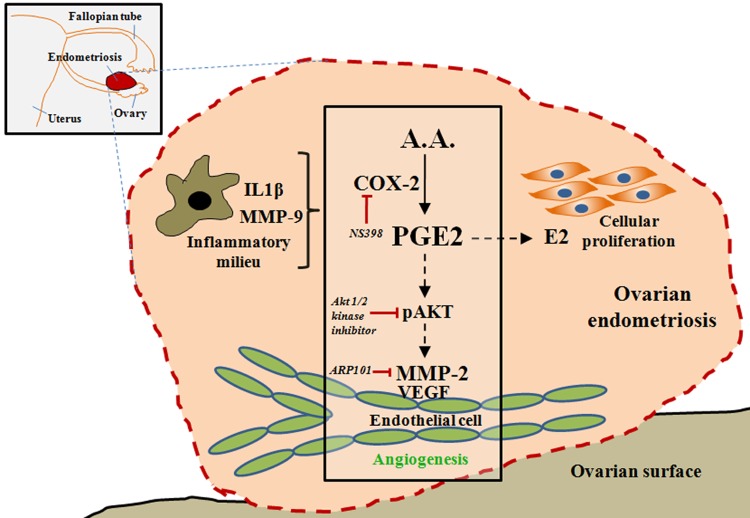
PGE2-mediated MMP-2 activity promotes angiogenesis in endometriosis. Endometriosis is an inflammatory, proliferative disease with increased PGE2 responses. The production of PGE2 from arachidonic acid (A.A.) is regulated by cycloxygenase-2 (COX-2). The present study found that the COX-2-PGE2-pAKT axis is involved in regulation of MMP-2 activity in endothelial cells which in turn promote angiogenesis in endometriosis.

CAM (*in ovo*) assay shows the real time angiogenesis process as compared to endothelial tube formation and was performed to validate the angiogenic roles of MMP-2. Implantation of gelatin disc containing human endometriotic extract elevated angiogenic branching in CAM assay, suggesting pro-angiogenic niche in endometriosis. Treatment with MMP-2 inhibitor in the same implant attenuated angiogenic branching confirming the necessity of MMP-2 in the process of angiogenesis. In murine model of endometriosis, inhibition of MMP-2 exhibited decreased numbers of endometriotic lesions along with reduced VEGF expressions, suggesting occurrence of endometriosis and associated angiogenesis through MMP-2 activities. Furthermore, treatment with celecoxib suppressed endometriotic lesion by reducing MMP-2 activities via inhibition of pAKT pathway. Our study reveals the involvement of COX-2-PGE2-pAKT-mediated signaling pathways in regulation of MMP-2 activity and subsequent angiogenesis in endometriosis (Fig 9). Apart from the PGE2-mediated MMP-2 activity, MMPs can also influence angiogenesis by several other mechanisms. MMPs are reported to promote the endothelial cell migration and tube formation by proteolytic cleavage of insoluble VEGF, FGF and chemo-tactic cryptic motifs of ECM components [35, 36]. Moreover, MMP-9-mediated activation of VEGF acts as a trigger for angiogenic responses during carcinogenesis[37]. MMP-2 is reported to induce VEGF expression through integrin αVβ3-mediated signaling pathway in cancer cells [20]. Until now few studies have documented regression of endometriosis upon total inhibition of MMPs in mouse model and CAM model of endometriosis [18, 38], however, we report for the first time the specific role of MMP-2 in endometriosis-associated angiogenesis.

In conclusion, ovarian endometriosis is associated with increased MMP-2 activity and pathological angiogenesis. PGE2, a pro-angiogenic factor for endometriosis, is involved in elevating MMP-2 activity in endothelial cells. Perturbation in the COX-2-PGE2-pAKT axis attenuates MMP-2 activity as well as endothelial tube formation. MMP-2 is involved in cellular migration and invasion, thus selective inhibition of MMP-2 activity significantly impedes tube formation as well as angiogenesis. Our data suggest the role of MMP-2 through PGE2-mediated pathway for the promotion of angiogenesis in endometriosis.

## Supporting Information

S1 Fig Original gel pictures of western blottings from human study. Western blot for VREGFR2 (A), VEGF (B), MT1MMP (C) and TIMP-2 (D) for ectopic samples of different stages of ovarian endometriosis.
(TIF)

S2 Fig Original gel pictures of gelatin zymography from human study. Stage dependent study of ectopic ovarian endometriosis samples (A). Serum data for control and endometriosis patients in a stage dependent manner (B). Zymography performed from eutopic endometrium of women with and without endometriosis (C).
(TIF)

S3 Fig Original gel pictures of western blotting and zymography from *in vitro* study. Studies on MMP-2 activities for dose dependent effect of PGE2 on HUVEC cells (A) and western blotting (B). Evaluation of MMP-2 activities in mertigel assays (C).
(TIF)

S4 Fig Original gel pictures of western blotting and zymography from *in vivo* study. Evaluation of MMP-2 activities for mouse model of endometriosis (n = 4) and effect of MMP-2i and COX-2i thereon through zymography (A) and western blotting (B).
(TIF)

S1 Table Details of the antibodies used during the experiments. (DOC)

## References

[1] Vercellini P, Vigano P, Somigliana E, Fedele L. Endometriosis: pathogenesis and treatment. Nat Rev Endocrinol. 2014;10(5):261–75. Epub 2013/12/25. 10.1038/nrendo.2013.255 . 24366116

[2] Bulun SE, Zeitoun K, Takayama K, Noble L, Michael D, Simpson E, et al Estrogen production in endometriosis and use of aromatase inhibitors to treat endometriosis. Endocr Relat Cancer. 1999;6(2):293–301. Epub 2000/03/24. 10.1677/erc.0.0060293 . 10731122

[3] Rocha AL, Reis FM, Taylor RN. Angiogenesis and endometriosis. Obstet Gynecol Int. 2013;2013:859619 Epub 2013/06/15. 10.1155/2013/859619 23766765 PMC3677669

[4] Carmeliet P, Jain RK. Angiogenesis in cancer and other diseases. Nature. 2000;407(6801):249–57. Epub 2000/09/23. 10.1038/35025220 . 11001068

[5] Potente M, Gerhardt H, Carmeliet P. Basic and therapeutic aspects of angiogenesis. Cell. 2011;146(6):873–87. Epub 2011/09/20. 10.1016/j.cell.2011.08.039 . 21925313

[6] Donnez J, Smoes P, Gillerot S, Casanas-Roux F, Nisolle M. Vascular endothelial growth factor (VEGF) in endometriosis. Hum Reprod. 1998;13(6):1686–90. Epub 1998/08/04. 10.1093/humrep/13.6.1686 . 9688413

[7] McLaren J. Vascular endothelial growth factor and endometriotic angiogenesis. Hum Reprod Update. 2000;6(1):45–55. Epub 2000/03/11. 10.1093/humupd/6.1.45 . 10711829

[8] McLaren J, Prentice A, Charnock-Jones DS, Millican SA, Muller KH, Sharkey AM, et al Vascular endothelial growth factor is produced by peritoneal fluid macrophages in endometriosis and is regulated by ovarian steroids. J Clin Invest. 1996;98(2):482–9. Epub 1996/07/15. 10.1172/jci118815 8755660 PMC507453

[9] Rocha AL, Reis FM, Taylor RN. Angiogenesis and endometriosis. Obstet Gynecol Int. 2013;859619(10):26. 10.1155/2013/859619 PMC3677669 23766765

[10] Brinckerhoff CE, Matrisian LM. Matrix metalloproteinases: a tail of a frog that became a prince. Nat Rev Mol Cell Biol. 2002;3(3):207–14. 10.1038/nrm763 11994741

[11] Page-McCaw A, Ewald AJ, Werb Z. Matrix metalloproteinases and the regulation of tissue remodelling. Nat Rev Mol Cell Biol. 2007;8(3):221–33. 10.1038/nrm2125 17318226 PMC2760082

[12] Radisky DC, Levy DD, Littlepage LE, Liu H, Nelson CM, Fata JE, et al Rac1b and reactive oxygen species mediate MMP-3-induced EMT and genomic instability. Nature. 2005;436(7047):123–7. Epub 2005/07/08. 10.1038/nature03688 16001073 PMC2784913

[13] Brew K, Nagase H. The tissue inhibitors of metalloproteinases (TIMPs): An ancient family with structural and functional diversity. Biochim Biophy Acta. 2010;1803(1):55–71. 10.1016/j.bbamcr.2010.01.003 . PMC2853873 20080133

[14] Osteen KG, Yeaman GR, Bruner-Tran KL. Matrix metalloproteinases and endometriosis. Semin Reprod Med. 2003;21(2):155–64. 10.1055/s-2003-41322 12917785

[15] Paul S, Sharma AV, Mahapatra PD, Bhattacharya P, Reiter RJ, Swarnakar S. Role of melatonin in regulating matrix metalloproteinase-9 via tissue inhibitors of metalloproteinase-1 during protection against endometriosis. J Pineal Res. 2008;44(4):439–49. Epub 2008/02/27. 10.1111/j.1600-079X.2007.00547.x . 18298469

[16] Paul S, Bhattacharya P, Das Mahapatra P, Swarnakar S. Melatonin protects against endometriosis via regulation of matrix metalloproteinase-3 and an apoptotic pathway. J Pineal Res. 2010;49(2):156–68. Epub 2010/07/09. 10.1111/j.1600-079X.2010.00780.x . 20609072

[17] Jana S, Paul S, Swarnakar S. Curcumin as anti-endometriotic agent: implication of MMP-3 and intrinsic apoptotic pathway. Biochem Pharmacol. 2012;83(6):797–804. Epub 2012/01/10. 10.1016/j.bcp.2011.12.030 . 22227273

[18] Bruner KL, Matrisian LM, Rodgers WH, Gorstein F, Osteen KG. Suppression of matrix metalloproteinases inhibits establishment of ectopic lesions by human endometrium in nude mice. J Clin Invest. 1997;99(12):2851–7. 10.1172/JCI119478 9185507 PMC508135

[19] Iurlaro M, Loverro G, Vacca A, Cormio G, Ribatti D, Minischetti M, et al Angiogenesis extent and expression of matrix metalloproteinase-2 and -9 correlate with upgrading and myometrial invasion in endometrial carcinoma. Eur J Clin Invest. 1999;29(9):793–801. 10.1046/j.1365-2362.1999.00532.x 10469168

[20] Chetty C, Lakka SS, Bhoopathi P, Rao JS. MMP-2 alters VEGF expression via alphaVbeta3 integrin-mediated PI3K/AKT signaling in A549 lung cancer cells. Int J Cancer. 2010;127(5):1081–95. 10.1002/ijc.25134 20027628 PMC2891576

[21] Li ZG, Lang JH, Leng JH, Liu DY. Increased levels of prostaglandin E2 and bcl-2 in peritoneal fluid and serum of patients with endometriosis. Zhonghua Fu Chan Ke Za Zhi. 2005;40(9):598–600. Epub 2005/10/06. . 16202314

[22] Huang H-F, Hong L-H, Tan Y, Sheng J-Z. Matrix metalloproteinase 2 is associated with changes in steroid hormones in the sera and peritoneal fluid of patients with endometriosis. Fertil Steril. 2004;81(5):1235–9. 10.1016/j.fertnstert.2003.10.027. 10.1016/j.fertnstert.2003.10.027 15136083

[23] Kinoshita T, Sato H, Okada A, Ohuchi E, Imai K, Okada Y, et al TIMP-2 promotes activation of progelatinase A by membrane-type 1 matrix metalloproteinase immobilized on agarose beads. J Biol Chem. 1998;273(26):16098–103. Epub 1998/06/20. 10.1074/jbc.273.26.16098 . 9632662

[24] Zhou Z, Apte SS, Soininen R, Cao R, Baaklini GY, Rauser RW, et al Impaired endochondral ossification and angiogenesis in mice deficient in membrane-type matrix metalloproteinase I. Proc Natl Acad Sci U S A. 2000;97(8):4052–7. Epub 2000/03/29. 10.1073/pnas.060037197 10737763 PMC18145

[25] Brosens I, Brosens JJ, Benagiano G. The eutopic endometrium in endometriosis: are the changes of clinical significance? Reprod Biomed Online. 2012;24(5):496–502. 10.1016/j.rbmo.2012.01.022 22417665

[26] Chakraborti S, Mandal M, Das S, Mandal A, Chakraborti T. Regulation of matrix metalloproteinases: an overview. Mol Cell Biochem. 2003;253(1–2):269–85. 14619979 10.1023/a:1026028303196

[27] Karck U, Reister F, Schafer W, Zahradnik HP, Breckwoldt M. PGE2 and PGF2 alpha release by human peritoneal macrophages in endometriosis. Prostaglandins. 1996;51(1):49–60. Epub 1996/01/01. 10.1016/0090-6980(95)00159-x . 8900443

[28] Sacco K, Portelli M, Pollacco J, Schembri-Wismayer P, Calleja-Agius J. The role of prostaglandin E2 in endometriosis. Gynecol Endocrinol. 2012;28(2):134–8. Epub 2011/10/19. 10.3109/09513590.2011.588753 . 22003899

[29] Ferrara N. VEGF and the quest for tumour angiogenesis factors. Nat Rev Cancer. 2002;2(10):795–803. 10.1038/nrc909 12360282

[30] Olsson AK, Dimberg A, Kreuger J, Claesson-Welsh L. VEGF receptor signalling—in control of vascular function. Nat Rev Mol Cell Biol. 2006;7(5):359–71. 10.1038/nrm1911 16633338

[31] Fagotti A, Ferrandina G, Fanfani F, Legge F, Lauriola L, Gessi M, et al Analysis of cyclooxygenase-2 (COX-2) expression in different sites of endometriosis and correlation with clinico-pathological parameters. Human Reprod. 2004;19(2):393–7. 10.1093/humrep/deh054 14747187

[32] Jana S, Rudra DS, Paul S, Snehasikta S. Curcumin delays endometriosis development by inhibiting MMP-2 activity. Indian J Biochem Biophys. 2012;49(5):342–8. 23259320

[33] Bernardo MM, Fridman R. TIMP-2 (tissue inhibitor of metalloproteinase-2) regulates MMP-2 (matrix metalloproteinase-2) activity in the extracellular environment after pro-MMP-2 activation by MT1 (membrane type 1)-MMP. Biochem J. 2003;374(Pt 3):739–45. 10.1042/bj20030557 . 12755684 PMC1223627

[34] Seo DW, Li H, Guedez L, Wingfield PT, Diaz T, Salloum R, et al TIMP-2 mediated inhibition of angiogenesis: an MMP-independent mechanism. Cell. 2003;114(2):171–80. Epub 2003/07/31. . 12887919 10.1016/s0092-8674(03)00551-8

[35] Contois L, Akalu A, Brooks PC. Integrins as “functional hubs” in the regulation of pathological angiogenesis. Semin Cancer Biol. 2009;19(5):318–28. 10.1016/j.semcancer.2009.05.002 19482089 PMC2806796

[36] Deryugina EI, Quigley JP. Pleiotropic roles of matrix metalloproteinases in tumor angiogenesis: Contrasting, overlapping and compensatory functions. BBA-Mol Cell Res. 2010;1803(1):103–20. 10.1016/j.bbamcr.2009.09.017. 19800930 PMC2824055

[37] Bergers G, Brekken R, McMahon G, Vu TH, Itoh T, Tamaki K, et al Matrix metalloproteinase-9 triggers the angiogenic switch during carcinogenesis. Nat Cell Biol. 2000;2(10):737–44. 10.1038/35036374 11025665 PMC2852586

[38] Nap AW, Dunselman GA, de Goeij AF, Evers JL, Groothuis PG. Inhibiting MMP activity prevents the development of endometriosis in the chicken chorioallantoic membrane model. Hum Reprod. 2004;19(10):2180–7. 10.1093/humrep/deh408 15242997

