# Supplementary figures and images for: Regulation of Matrix Metalloproteinase-2 Activity by COX-2-PGE2-pAKT Axis Promotes Angiogenesis in Endometriosis
Source: PLoS One. 2016 Oct 3;11(10):e0163540. doi: 10.1371/journal.pone.0163540 (PMC5047632; doi:10.1371/journal.pone.0163540)

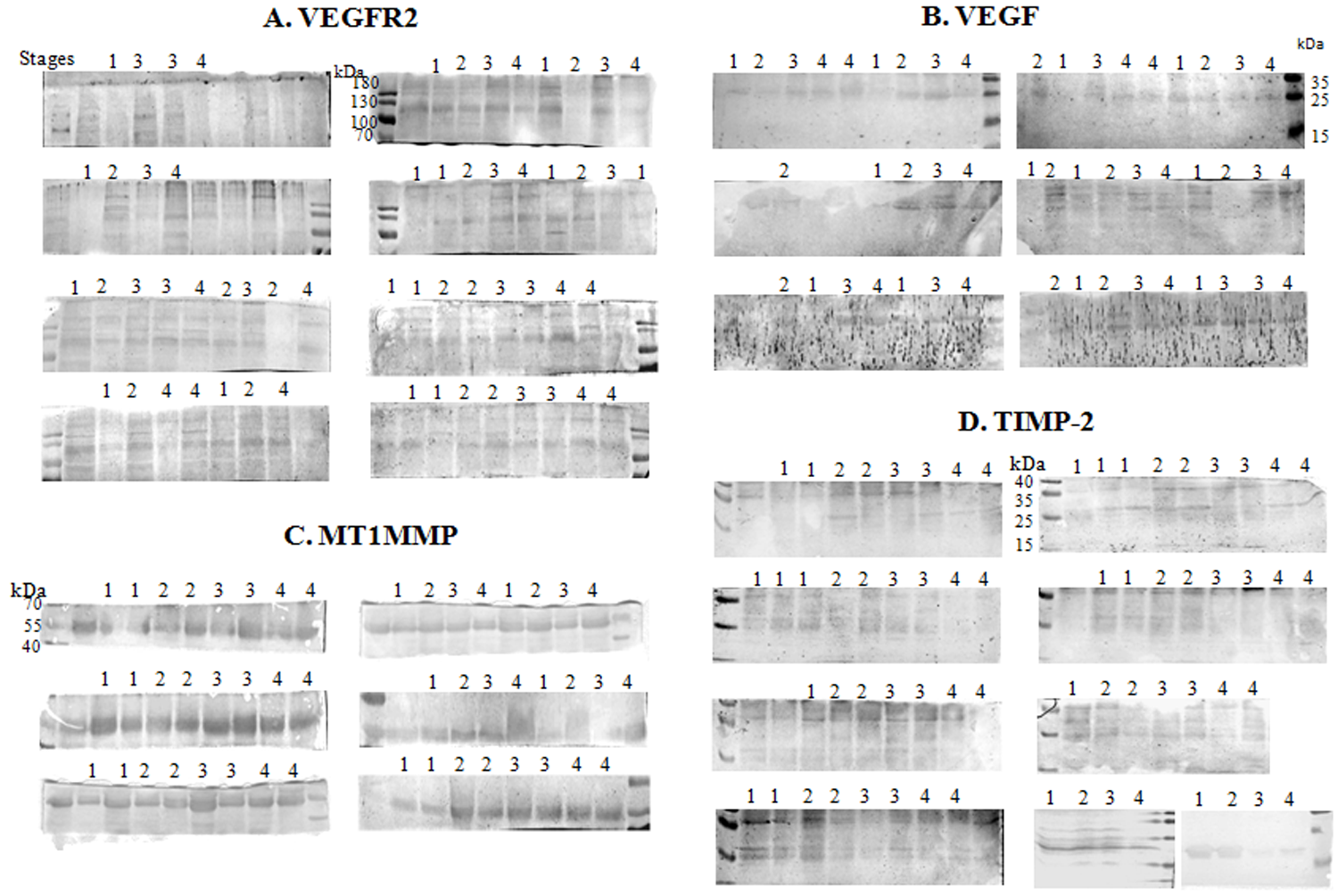

Supplement: S1 Fig — Western blot for VREGFR2 (A), VEGF (B), MT1MMP (C) and TIMP-2 (D) for ectopic samples of different stages of ovarian endometriosis. (TIF) [file pone.0163540.s001.tif]

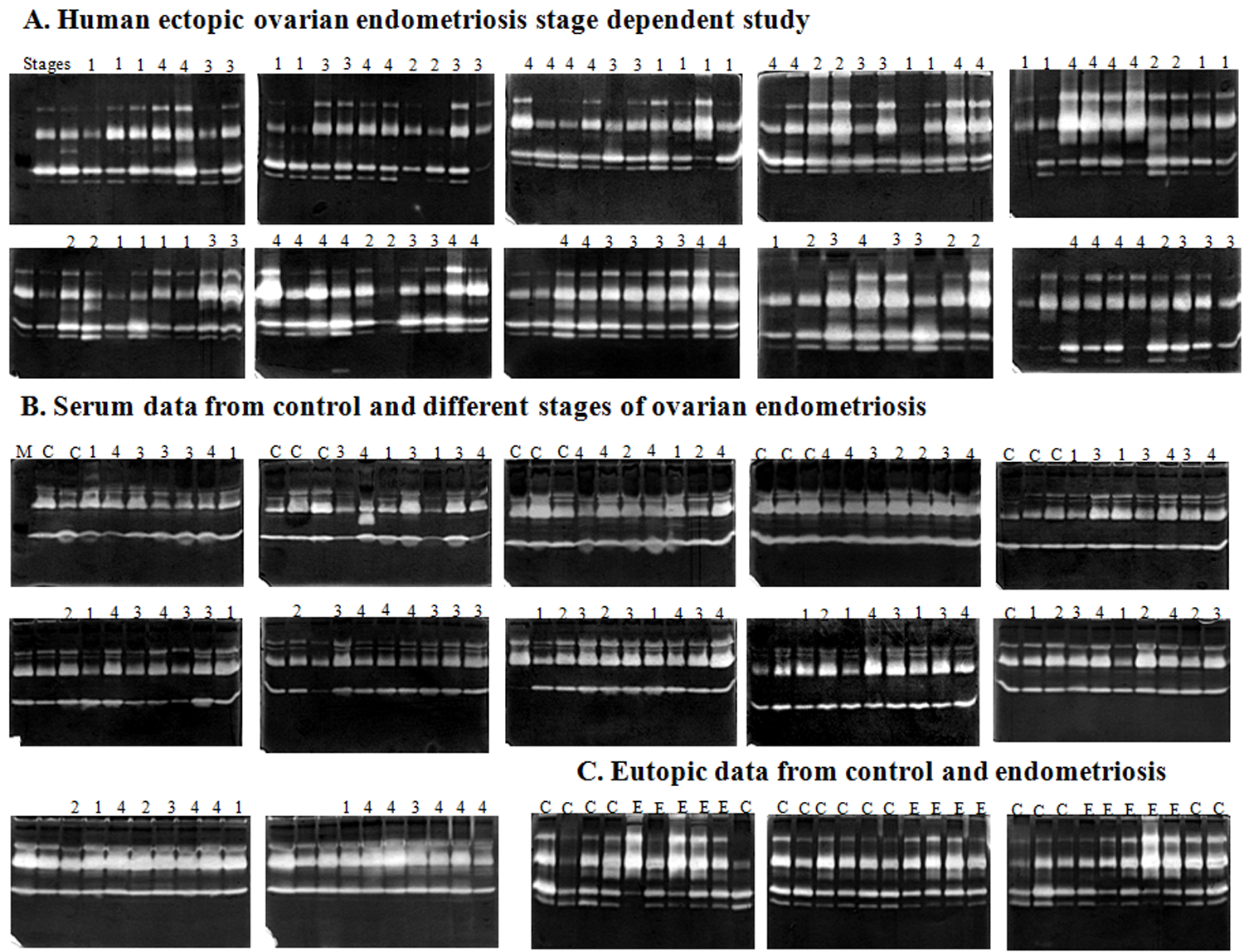

Supplement: S2 Fig — Stage dependent study of ectopic ovarian endometriosis samples (A). Serum data for control and endometriosis patients in a stage dependent manner (B). Zymography performed from eutopic endometrium of women with and without endometriosis (C). (TIF) [file pone.0163540.s002.tif]

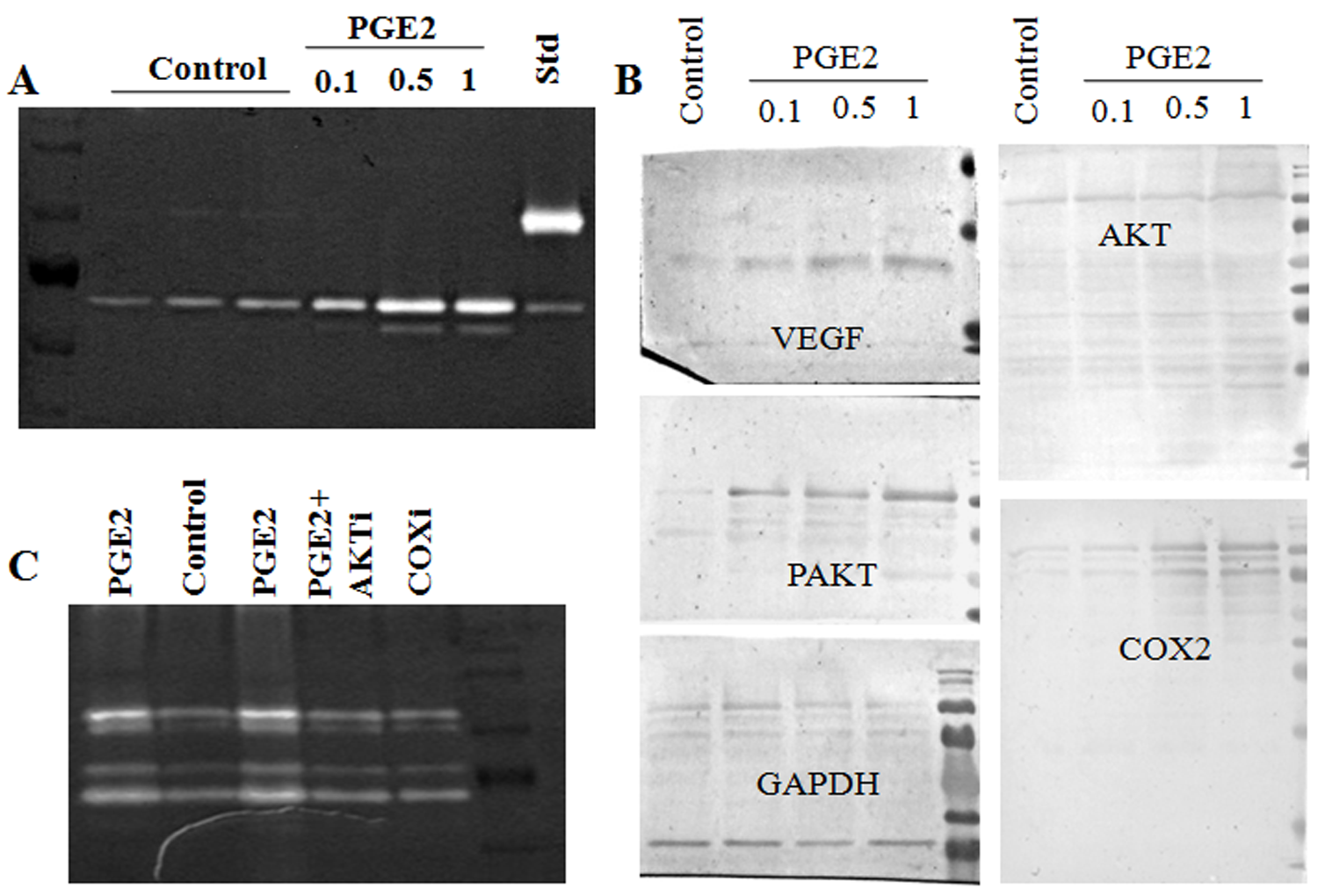

Supplement: S3 Fig — Studies on MMP-2 activities for dose dependent effect of PGE2 on HUVEC cells (A) and western blotting (B). Evaluation of MMP-2 activities in mertigel assays (C). (TIF) [file pone.0163540.s003.tif]

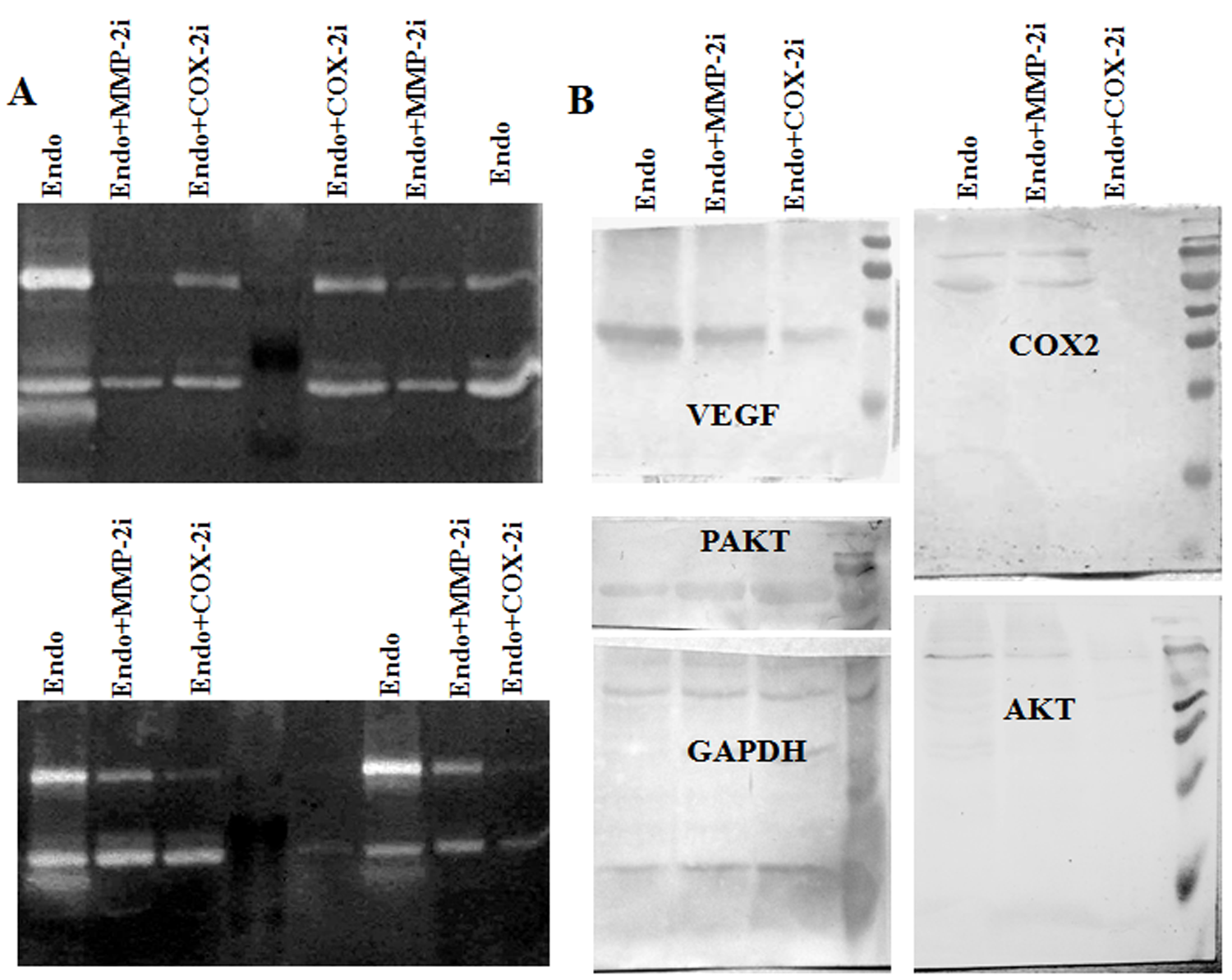

Supplement: S4 Fig — Evaluation of MMP-2 activities for mouse model of endometriosis (n = 4) and effect of MMP-2i and COX-2i thereon through zymography (A) and western blotting (B). (TIF) [file pone.0163540.s004.tif]
