# Supplementary material for: Regulation of Matrix Metalloproteinase-2 Activity by COX-2-PGE2-pAKT Axis Promotes Angiogenesis in Endometriosis
Source: PLoS One. 2016 Oct 3;11(10):e0163540. doi: 10.1371/journal.pone.0163540 (PMC5047632; doi:10.1371/journal.pone.0163540)
Supplement: S1 Table — (DOC) [file pone.0163540.s005.doc]

**S1 Table. Details of the antibodies used during the experiments.**

| **Name** | **Company** | **Catalogue** | **Host species** | **Final dilution** | **Antibody ID** |
| --- | --- | --- | --- | --- | --- |
| vWF | Santa Cruz Biotechnology | SC-8068 | Goat polyclonal IgG | 1:100 | [AB_2216590](http://antibodyregistry.org/AB_2216590) |
| COX-2 | Santa Cruz Biotechnology | SC-1745 | Goat polyclonal IgG | 1:100 | [AB_631309](http://antibodyregistry.org/AB_631309) |
| TIMP-2 | Santa Cruz Biotechnology | SC-9905 | Goat polyclonal IgG | 1:500 | [AB_2303478](http://antibodyregistry.org/AB_2303478) |
| MT1MMP | Santa Cruz Biotechnology | SC-12366 | Goat polyclonal IgG | 1:500 | [AB_2303478](http://antibodyregistry.org/AB_2303478) |
| VEGF | Santa Cruz Biotechnology | SC-507 | Rabbit polyclonal IgG | 1:500 | [AB_2212666](http://antibodyregistry.org/AB_2212666) |
| VERGFR2/Flk1 | Santa Cruz Biotechnology | SC-504 | Rabbit polyclonal IgG | 1:500 | [AB_632600](http://antibodyregistry.org/AB_632600) |
| pAKT | Santa Cruz Biotechnology | SC-16646R | Rabbit polyclonal IgG | 1:500 | [AB_667742](http://antibodyregistry.org/AB_667742) |
| AKT | Santa Cruz Biotechnology | SC-8312 | Rabbit polyclonal IgG | 1:500 | [AB_671714](http://antibodyregistry.org/AB_671714) |
| GAPDH | Santa Cruz Biotechnology | SC-48167 | Goat polyclonal IgG | 1:500 | [AB_1563046](http://antibodyregistry.org/AB_1563046) |
